# On Provisional Callus in Fractured Bones

**Published:** 1853-02

**Authors:** Frank H. Hamilton

**Affiliations:** Professor of Surgery in the University of Buffalo, and one of the Surgeons to the Buffalo Hospital


BUFFALO MEDICAL JOURNAL

AND

MONTHLY REVIEW.

VOL. 8. FEBRUARY, 1853. NO. 9.

### ORIGINAL COMMUNICATIONS.

ART. I. — *On Provisional Callus in Fractured Bones.* By FRANK H. HAMILTON, A. M., M. D., Professor of Surgery in the University of Buffalo, and one of the Surgeons to the Buffalo Hospital.

DR. FLINT:

You are aware that nearly a year since I called your attention to my views upon the subject of the union of broken bones, and that I then regarded these views as original and as entertained only by myself. In May last I prepared the following paper as a contribution to the semi-annual meeting of the State Medical Society, but learning soon after that I had been anticipated by Mr. Paget, of London, I withheld the paper from the Society, giving to its publication an indefinate, if not final postponement.

I have now, however, determined to publish it, and in the same form in which it was originally written, before I had seen or heard of the views of Mr. Paget.

My reasons for this determination you will permit me to state:

James Paget is the Professor of Anatomy and Surgery to the Royal College of Surgeons of England, and the exposition of his views of the union of broken bones constituted a portion of a series of lectures on the "Processes of Repair and Reproduction after Injuries," delivered by him before the College three years since. These lectures were published in the Medical Gazette for 1849, and subsequently in "Ranking's Abstract" for 1850.

It would seem that the position and rank of the author ought to have insured to his new doctrines general attention among surgeons and pathologists on both sides of the Atlantic. Yet I doubt whether they have obtained more than a casual and scarcely a respectful attention either here or elsewhere. I have seen no notice of them in American Journals, and among the many eminent surgeons with whom I have conversed, both teachers and practitioners, only one or two retained a vague impression of their existence. By a letter also from a friend in Paris, I learn that upon careful inquiry the writer could not ascertain that any one in that city had seen or heard of the doctrines announced by Paget; and the only evidence I have that these doctrines have attracted any more attention at home, in great Britain, than they have abroad, is furnished in the notice given of them by Pirriè, the Professor of Surgery in the University of Aberdeen, in his work on surgery published during the present year. After having explained the mode of union of bones, as taught by Dupuytren, he remarks:

"Such are the views of Dupuytren on this interesting subject; and until lately they were generally received as the correct explanation of the successive changes that take place, both in man, and in the lower animals, from the occurrence of fracture until the injury is completely repaired.

"Mr. Paget, in his 'Lectures on Repair and Reproduction after Injuries,' has brought forward different views from those which formerly prevailed regarding the repair of a fractured human bone, and has supported his opinions by most conclusive evidence. His views on this subject are in accordance with those of Mr. Stanley."—Pirriè's Surgery, p. 127.

[I think Mr. Pirriè is not entirely right in saying that Mr. Paget's views "are in accordance with those of Mr. Stanley." Mr. Stanley says, "in the human subject, no such cartilaginous and osseous deposit uniformly takes place around the fractured bone; here, therefore, it is not an essential part of the reparative process." This passage implies that he regards provisional callus as a general but "not uniform" occurrence; useful, but not "essential." These are by no means in accordance with Mr. Paget's views; nor are they the views which I propose to advocate. If such were actually Mr. Paget's views, then it will be seen that we are not agreed, and I should still claim originality for my doctrine.]

I thought it proper, therefore, that I should attempt to direct the attention of my professional brethren to Mr. Paget, by a reproduction of his and by a public statement of my own conclusions — conclusions nearly or quite identical, to which, without concert, we had almost simultaneously arrived, and yet by somewhat different roads.

Mr. Paget's first impressions of the fallacies of the doctrines of Dupuytren, and his subsequent full convictions were obtained solely from pathological specimens — from "the large collection of fractures in the museum of the college." While my first impressions were received from examinations of the progress of union in the living subject, and by similar examinations often repeated, have my early impressions grown into mature convictions.

I cannot but believe that an experience which, as will be seen by my "Fracture Tables," lately published by Mr. Boardman, now includes a personal examination upon the living subject, of nearly six hundred fractures, will possess some value as corroborative testimony.

In this way, mainly, have I arrived at my conclusions, yet I have not neglected the examination also of pathological specimens, such as I could find in my own or other private or public museums.

In 1844 I studied very critically the famous Dupuytren Museum, at Paris, which is peculiarly rich in fractures; and the results of my study and analysis were subsequently published in a tabular form in the Buffalo Medical Journal for 1849. And wherever else upon the continent, in Great Britain or in this country similar collections came within my reach, I have never failed to avail myself of them for the purposes of study. Thus from many and various sources the evidence has been derived, and the conviction has been gathering upon me that Dupuytren had greatly misapprehended the process of union of broken bones in the human subject: and this evidence was no less abundant in his own beautiful collection at Paris than elsewhere.

It is true that I was then especially directing my observations to the existence of deformity or perfection in the union of bones, and their relative frequency. But the frequent — I might almost say, constant absence of provisional callus in bones well united, and especially of the outer ring or "ferrule," very early attracted my notice and excited my surprise: and when afterward I had collected the undeniable proof that in a large majority of cases of fracture the union occurs by overlapping, there came also the very natural suggestion that very often, no doubt, the surgeon mistook this overlapping for provisional callus. That surgeons do often commit this error, I have since confirmed by my own observations in a multitude of cases.

Trusting that the care and attention which I have bestowed upon this subject entitle me to speak in a degree authoritatively, I have given you this paper containing my own views, for publication; and I have also to request that you will allow it to be accompanied with the able lecture of Mr. Paget, as delivered before the Royal College of London.

Yours truly, FRANK H. HAMILTON.

*Provisional Callus.*

While prosecuting my investigations for the purpose of ascertaining the "average results in the treatment of fractures," several other points of interest have been suggested, some of which I have sought carefully to determine. That which arrested my attention earliest, and which I have most attentively noticed all along, until I have at length reached a satisfactory conclusion, is *the almost constant absence of provisional callus, both during the process of cure, and in the result, where the fractured ends have been kept in tolerable apposition, and free from undue excitement.*

I had never doubted before that provisional callus would be found in all cases of union of broken bones where the health of the patients and the condition of the fragments permitted the restoration to proceed in a natura manner. It was this which the experiments of Du Hamel and of Dupuytren seemed to have established.

The old surgeons spoke of an "exuberance" of callus, or of a "redundance," occurring as the result of displacement, frequent disturbance, inflammation, &c., but they never spoke of it as constituting any part of a healthy, normal process. Thus Mr. Pott writes:

"When a bone has been broken transversely, or nearly so, and its inequalities are therefore neither many nor great, when such broken parts have been happily and properly coapted, and proper methods have been used to keep them constantly and steadily in such state of coaptation, the divided parts unite by the intervention of the circulating juice, just as the softer parts do, allowing a difference of space of time for different texture and consistence. When the union of a broken bone under such circumstances has been procured, the place where such union has been made will be very little perceptible; it will be no deformity, nor will it occasion any inconvenience. It will indeed be discoverable, like a cicatrix of a wound in a softer part; but there will be no redundance of callus, because none will be wanted."—*Pott's Surg.; First Amer. Ed., vol. 1, p.* 234.

It was believed that in refuting these erronious doctrines of Mr. Pott, and of his cotemporaries, Dupuytren had performed an important service, and had made a most valuable contribution to surgical pathology. Mr. Samuel Cooper congratulating the art upon these modern discoveries speaks after this manner:

"A few years ago lecturers on surgery got over this subject very easily, and those teachers, whom I happened to attend, explained the matter in a concise and summary way, by stating, the only difference between the union of bone and that of soft parts, was that the coagulating lymph, effused between the ends of fracture, gradually acquired the consistence of cartilage, earthy matter was deposited in it, and thus the bone was united, and acquired its former strength, the only particularity being in fact the deposit of the phosphate of lime in the uniting medium." — *Saml. Coop. First Lines; Fourth Amer. Ed., vol. I, p.* 282.

Although it has not escaped the observation of many shrewd writers that Dupuytren's experiments were all made upon brute animals, and they have therefore received with a prudent caution many of his conclusions, such as the period of time occupied in the several stages of reparation, the sources of the callus, &c., yet has it seldom if ever happened that they have called in question, or expressed a doubt of the accuracy of his conclusions as to the main point, viz., the existence of a provisional callus as a temporary bond of union in all cases where bones unite by a natural and undisturbed process.

I have looked carefully for such doubts or denials, but I find nothing of the kind clearly expressed; that is, nothing which can be construed into a substantial doubt or a denial that broken bones unite naturally through the interposition of provisional callus. Mr. Liston, in the following paragraph, speaks like one who sought to reconcile his own observations, not yet reduced to a system, with certain conflicting, but everywhere established doctrines, the correctness of whose maxims it would, perhaps, be scarcely respectable for a man of science to call in question:

"Union of divided bones, as of soft parts, is preceded by incited circulation in the part and effusion of matter. The extent of action is regulated by that of the injury, whether inflicted by accident or by operation. If the soft parts have not been much bruised, if the bone and its covering are merely separated and slightly displaced, and then speedily put in contact, *the incited action and the effusion are limited to the divided parts. There is no irregularity afterward at the point of fracture*, the new matter that is not required being absorbed soon after deposition; the bone is smooth and even as before. If, on the contrary, there is much displacement, and if that is not entirely removed, intense action ensues both in the soft and hard parts, there is great effusion of new matter or *callus*."

It is obvious, I think, that Mr. Liston had noticed the absence of provisional callus in simple and well-adjusted fractures, at least *soon* after the union was completed; a fact for which he offers the usual explanation, viz., that it is absorbed *soon after* deposition: yet he does not recognize its inconsistency with his preeeding statement that in such simple fractures the "effusion is limited to the divided parts." But the fact that he had not noticed the presence of provisional callus even during the progress of restoration, seems probable from his account of what occurs in the opposite class of cases, where

"displacement," &c., exist, for there is now, he informs us, "great effusion of *new* matter or *callus*." The term *callus* or *new* matter being here first employed.

Perhaps I am disposed to infer too much from this somewhat ambiguous paragraph, but the language will certainly admit of the construction which I have given it, and I cannot dispossess myself of the belief that this great and eminently practical surgeon had seen and noticed much to conflict with the views of Dupuytren.

Mr. Stanley has made the nearest approach to a repudiation of the doctrines of Dupuytren of any man whose writings I have seen. In the preface to his excellent "Treatise on the Diseases of Bones," occurs the following passage:

"Experiments in animals have not accomplished so much for the elucidation of the reparative process of bone in man as might probably have been expected. The circumstances attendant on the fractured or necrosed bone, in man, are essentially different from those of the experiment of breaking, or causing the death of a bone in animals. Thus around the fractured bone of an animal, the deposit of cartilaginous and osseous substance, which has been designated provisional callus, is of uniform occurrence. But, in the human subject, no such cartilaginous and osseous deposit uniformly takes place around the fractured bone; here, therefore, it is not an essential part of the reparative process."

In this we have a plain declaration that provisional callus is not "uniformly" present in the union of divided bones, and that it is therefore "not an essential part of the reparative process."

But not in this admission of Mr. Stanley's, nor in the statements of any other writers do I find a complete expression of my own sentiments upon this subject.

I shall go much farther. I am now prepared confidently to affirm that the so-called provisional callus never constitutes any part of the reparative process in the union of divided bones, when all those circumstances of simplicity, apposition, quietude, health, just management, &c., obtain, which may properly be considered essential to a normal process — that bones unite most naturally by definitive callus, and that provisional callus is accidental and secondary — the result probably of undue excitement alone.

It may be, indeed, the rule that in the union of fractures some amount of provisional callus shall be found, but it will be because it is the rule rather than the exception that undue excitement exists. My fracture tables published a few years since, and farther observations lately made, will show that broken bones are seldom kept in such complete apposition as will allow nature to proceed without interruption or disturbance.

Permit me to state my belief in another form:

Broken bones unite when submitted to the most favorable circumstances, by definitive callus, or by a process allied to adhesion — by *first intention:* but under less favorable circumstances by provisional callus, or by a process allied to granulation — by *second intention.*

The venerable and distinguished Dr. Mussy, of Cincinnati, to whom I stated my views, and by whom I believe their general correctness is admitted, said that he would express his notions of the reparation of fractured bones by saying, that nature, or the Great Author of nature, first sought to repair the injury in the simplest possible manner, by a direct union of the ends of the bones; but being defeated in this, she then chose the next best alternative, viz., to form a temporary callus, and this was the origin and object of nature's splint.

To these views, with certain qualifications, I assent. But then provisional callus is no longer the normal, but only a contingent or alternative process.

Such are the conclusions to which I have arrived, after having examined several hundred fractures, nearly one-half of which were sufficiently recent to have enabled me to have discovered the callus if any had ever existed.

There is generally no difficulty in determining the presence of provisional callus in fractures of such superficial bones as the inferior maxilla, clavicle, radius, ulna and tibia, of the metacarpal, metatarsal and phalangeal bones, and indeed very often in fractures of other bones. Frequently the swelling is so inconsiderable that the surface of the bones can be distinctly felt at any period of the process of union. I have seized all such opportunities as were afforded me, and without being able to state numerically the result I have no remaining doubt that provisional callus is not present in any stage of the reparation where the conditions of health, &c., &c., before stated, exist.

The accuracy of these conclusions can only be tested by similar examinations upon the dead or living human subject. It is not possible, I think, to put the limb of any brute animal into that condition of rest requisite to determine nature's first intention: and here is the source of the fallacy into which Dupuytren and his disciples have been lead.

While my convictions upon this subject have originated and been confirmed by my own observations, I find also many substantial collateral evidences which cannot properly be overlooked in the argument.

If Mr. Stanley is correct in supposing that provisional callus is not essential to the process of union, and that it is not uniform in its occurrence, then it is reasonable to infer that this circumstance is not the first and established order of events: would bones, which are kept in exact apposition and undisturbed, unite by definitive callus alone, and that often in three or four weeks, if nature had established provisional callus as her chosen mode of union? If she is competent to unite bones by "first intention," why should she ever seek to unite by "second intention," unless driven to it as an alternative?

Nature is not so capricious. She never attempts to accomplish the same end, under the same circumstances, in different ways. To this law, I believe, there are no exceptions. It is clear, therefore, that Mr. Stanley admits too much or not enough.

I find another argument in support of my opinion in the fact that in the reparation of fractures occurring in certain bones, or in certain parts of bones, provisional callus, it is conceded, seldom or never occurs. Thus it is with the cranium, acromion process, coracoid, olecranon, patella, &c., and with all those portions of bones which are immediately invested with a synovial capsule.

If provisional callus is the established mode of reparation, why in these cases is it not furnished?

It was a very beautiful theory which referred the formation of provisional callus to an intelligent efficient cause, which in this manner sought to support the bones until a union of their divided ends was effected. Nor is the beauty of the conception marred by ascribing to it a more limited application, and invoking its interference only when the ordinary resources of nature have failed. We no longer hold that any such intelligent interposition is necessary in the *first* instance or in simple fractures, and if demanded at all, it is only for an exigency. But we have grave doubts whether nature ever allows any interference with her laws even in an exigency, unless by the substitution of a miracle. Provisional callus is just as much the inevitable result of natural laws, as is definitive callus, or any other reparative action. It is formed, because in that condition of the parts and of the general life, its formation was inevitable. Whether needed or not it will, under certain circumstances, exist.

It is affirmed, nevertheless, that in the fractures just named, this callus is not formed, because it is not required. While to me it seems that nowhere could it prove more useful, since, with the exception of the cranium, it is in these very cases that the obstacles to union are most numerous. In fractures of the patella, olecranon, &c., the action of the muscles tends constantly and powerfully to displace the fragments, and gladly would the surgeon avail himself of the assistance of a temporary callus, but it is rarely present, and then in no useful degree.

So, also, in fractures of the neck of the femur within the capsule, and in other similar cases, we cannot say that temporary callus would not be advantageous in facilitating the retention of the fragments, yet the "intelligent efficient agent" neglects to furnish it.

The only satisfactory reason which, as we think, can be assigned for the absence of callus in these cases, is found in the doctrines which I now advocate; that is to say, it is usually absent because that amount of excitement and irritation are usually absent which alone determine its formation. In the case of the olecranon, patella, &c., the fragments being separated from each other by muscular action, so that no painful pinchings or chafings occur, and their rough surfaces, or sharp points being rather drawn away from than protruded into the flesh, no sufficient provocation exists for the production of inflammation and effusion. Hence the failure of *provisional* callus, but wherever the fracture occurs, and however moderate the action, *definitive* callus does not fail; still the broken surfaces of the patella and olecranon are softened, and smoothed, and covered over with a new matter, which, if contact could have been secured and preserved, would certainly have served to consolidate and repair the breach. The natural reparative process proceeds, but only the accidental process is omitted. This latter, however, is seen again even here, when from other and unusual causes a sur-excitement is established.

Temporary callus is not formed upon bones invested with synovial membranes, because here, too, as in the neck of the femur, there are not so many structures lacerated and irritated, and the supply of this effusion must be the less not only in proportion to the less intensity of the inflammation, but also to the less amount of structures implicated.

Possibly other and more satisfactory reasons may be assigned why provisional callus is not formed usually when the neck of the femur is broken within the capsule; but we certainly can never admit the common, and as here applied, the too palpably absurd explanation, that it is not wanted. It is wanted! and in no case so much as now.

The same argument applies to fractures of the cranium. With less soft parts to suffer excitement and to determine effusion, and with no motion of the fragments to provoke it, provisional callus is less apt to occur. But you need not to be told, gentlemen, that here again, when the injury has been most severe and the consequent excitement most intense, the so-called "nature's splint," has been formed; although in this instance it could serve no possible purpose.

In short, provisional callus occurs still everywhere, when against and in the vicinity of the bone there is the requisite lesion and action; and it will occur as certainly when the fracture is incomplete, or when there is no fracture, but only a caries, a necrosis or a simple bony or even periosteal inflammation — and it becomes thus the basis of many tumors which grow from either the bone or the periosteum.

### *Recapitulation.*

*First.* Broken bones unite directly, naturally and by preference through the interposition of definitive callus.

*Second.* Broken bones unite indirectly, and accidentally, through the intervention of provisional callus.

*Third.* The absence of provisional callus does not denote that it could serve no useful purpose.

*Fourth.* Its presence does not indicate its necessity or utility.

*Fifth.* It has, therefore, no final purpose, but is the unavoidable result of a certain abnormal condition: and while it is doubtless true that in fractures it frequently renders valuable assistance to the surgeon, it is also equally true that it often proves a source of hindrance.

### *Extract from Prof. Paget's Fifth Lecture on the "Processes of Reproduction after Injuries."*

I shall not endeavor, in the present lecture, to treat fully of the Repair of Fractures. No one acquainted with the extent of the observations already made on this subject, and with the reputation of those who have been occupied with them, will blame me if I almost limit myself to the endeavor to explain only two or three points in the history of the repair of injured bones. The chief points that I have chosen are—first, the particulars in which the process of repair of fractures, observed in the human subject, deviates from that described from experiments upon lower animals; and secondly, the nature of the reparative material previous to its ossification.

On the first point, I must express my conviction that the description drawn by Dupuytren and others, from examinations of fractures in dogs, rabbits, birds, and other animals, cannot be applied without great deductions to the case of fractures in the human subject. True as the pictures are of the cases of the animals examined, they are exaggerations of the process in our own case. With a few exceptions, all that is written in these accounts of external and internal provisional and definitive callus, of the formations of cartilage and bone within the medullary tube and beneath the periosteum, can be traced only, as it were, in rudiment in the fractures of the human bones.

My impression of this was obtained while describing the large collection of fractures for the catalogue of the museum of the college.

With the concurrence of Mr. Stanley, who had long held a similar opinion,* I then wrote—"There is scarcely a specimen in the museum of such a provisional callus formed in the repair of a fractured human bone; in nearly every case of such fracture, the material of repair, whether cartilage or bone, is only inlaid between the broken surfaces, or between the adjacent parts of the fragments, and unites them by being fixed to both. In favorable conditions this appears to be the usual mode of repair, even though the fragments of the broken bone be very much displaced."

"But the formation of a provisional callus, completely encircling the broken ends and adjacent parts of the fragments, is usual in the repair of fractures of the bones of other mammalia, and of birds. * * * * * A similar but less perfect process is also shown in the accumulations of cartilage or bone which are often formed about fractures of the ribs, and of some other bones in the human subject, the fragments of which have not been held steady. It is probable, therefore, that the difference between the modes in which fractures are commonly united in man and other animals, respectively, depends in part on the movement to which the fragments are subjected in the latter; but probably in part, also, on the greater readiness with which, under all circumstances, bone is formed in the animals lower than man."†

Since that was written, I have examined many more specimens, and find the same rule true; namely, that in the ordinary repairs of simple fractures in the human subject, the reparative material, or callus, is merely inlaid between the several fragments; it fills up the interspaces between them and the angles at which one fragment overhangs another; but it does not encircle or ensheath them, in the manner implied in the description of provisional callus; nor is it in any considerable quantity, if at all, deposited either beneath the periosteum or within the medullary tube. In birds, dogs, and other ordinary subjects of experiments, the formation of a provisional, or as it may perhaps be better called, an *ensheathing* callus, is usual. It is illustrated by numerous specimens on the table; yet even in animals it is not constant. To obtain what would be called good specimens of provisional callus, the injuries must be inflicted upon young animals, and among these I cannot but suspect that particular instances have been selected for description—those in which less callus was formed having been put aside as imperfect instances of repair, though, in truth, they may have displayed the more natural process.

For fractures in the human subject, the evidence that union is accomplished by the reparative material being placed between, not within and around, the fragments—*i. e.*, as an intermediate, not an ensheathing callus—this evidence may be obtained by the examinations of such fractures even long after they are completely healed. In as many as you like to examine you will find the new bone formed exclusively between the fragments. Whether they were in apposition, or nearly so, or wide appart, still there is no appearance of new bone being formed on the outer side of any fragment—I mean on that side which is turned away from the other fragments. And this is the case even in those instances in which there is so much displacement of the fragments, and so much distortion, that we can hardly suppose the repair to have proceeded very quietly. Neither in any of these do you find new bone within

* Since the delivery of the lecture, Mr. Stanley has published his account of the Repair of Fractures in the descriptions of his beautiful "Illustrations of the Diseases of the Bones," p. 27.

† Pathological Catalogue of the Museum of the College of Surgeons, vol. ii, p. 37, the medullary tube. It may be objected by some to these specimens, that the fragments were once ensheathed and blocked up with callus, and that it has been since absorbed. But this is not probable, seeing that in many cases there remain, on the outer surfaces of the fragments, certain marks of their original form and slight irregularities. In one of the specimens which I present, we have traces of the healing of a long fissure, which appears now as a sunken groove, making it nearly certain that no new bone was formed over it. In another, is a detached piece of the wall of a femur turned quite round so that its periosteal surface lies on the periosteal surface of the principal fragment; yet, on the outer surface of this piece (which was the inner surface of its wall) the thin plates forming the boundary of the medullary tube are still unchanged.

But if any deem these and the like characters insufficient to prove the absence of ensheathing callus, and of callus extending into the medullary tube, yet recent specimens are not open to such doubts. I add, therefore, that (with the exceptions presently to be mentioned) in all the specimens of fracture that I have been able to examine, in the human subject, within six months of the time of the injury, there has been the same absence of provisional or ensheathing callus. The specimens here present are—a radius, four weeks after the fracture; another, four or five weeks; a tibia, five weeks; a femur, six weeks; another of the same date; a third, I should think, about eight or nine weeks; a radius, of somewhat later date; a tibia, eight weeks; a fibula, eleven weeks; a tibia, twelve weeks; and a tibia, sixteen weeks after the injury. Here are, also, others of various but unknown dates, all in process of apparently natural repair. All these were cases of simple fractures, and they include (with a few exceptions presently to be mentioned) all the specimens of such recent fractures, in the human subject, as are in the museums of the College and of St. Bartholomew's Hospital. The displacements and other conditions following the injury have been manifestly various: but all agree in this—that the fragments are united by intermediately-placed reparative substance, and that this, whether soft or osseous, in no case surrounds or ensheaths the fragments, or does more than just close in the medullary canal. When present in the largest quantity, it is only enough to smooth off the chief irregularities, and to fill up the interspaces and the angles or corners, between the fragments.

Such, then, appears to be the natural mode in which the reparative material is deposited for the union of fractures of human bones. And, regarding the particular position which it may in each case occupy, I do not know that it can be more exactly described, than by saying, that it is deposited where it is most wanted for the strengthening of the bone—so that, whatever would be the weak part of the bone, if unhealed, there is the new material placed, in quantity as well as position just adapted to the exigencies of the case, and restoring, as much as may be, the original condition and capacities of the bone.

If now it be inquired why this difference should exist in the corresponding processes in man and other animals, I believe still that it must be ascribed principally to the two causes already quoted from the catalogue—namely, the quietude in which fractures in our bones are maintained, and the naturally greater tendency to the production of new bone which animals always manifest. Even independently of surgery, in the case of fractures of the lower extremity, the human mode of progression almost compels a patient to take rest; and in fractures of the upper extremity, the circumstances of human life and society permit him to do so far more than other animals can. The whole process of repair is, therefore, more quietly conducted; and, as we may say, there is comparatively little need of the strength which the formation of provisional callus would give a broken limb.

The exception to the rule of difference in the repair of human bones and those of animals confirm it as thus explained; for the only bones in which, in the human subject, a provisional callus is generally or naturally formed, for the repair of fractures, are the ribs. In cases of fractured ribs one may see, indeed, a very close imitation of that which is described, from experiments on animals, as the ordinary mode of union. The provisional callus is well formed under the periosteum, and encircles, like a broad ring or ferrule, both the fragments, and may almost completely ossify before their union is accomplished, or even apparently begun.

Another bone for the repair of which, but more rarely, callus is formed around the ligaments, is the clavicle; and the best specimen in which I have here seen it is one in which the fracture was not detected, and the fragments were allowed to move on one another, till the patient died twelve weeks after the injury.

Except in such cases as these of fractures not kept at rest, I doubt whether a natural formation of callus beneath the periosteum, or within the medullary tube of a human bone, would ever occur. In disease, the occurrence is not so rare; for, when the natural process of union fails altogether, the loose ends of the bones may be inclosed within a case formed wholly or in part of bone; or an imitation of callus may be made by a gradual morbid accumulation of bone around a fracture, even after its natural union.

But I think the comparative restlessness of animals is not alone sufficient to account for all the difference in the processes. The remainder may be ascribed to their greater tendency, in all circumstances, to the formation of new bone. Not in fractures alone, but in necrosis, this is shown. It is very rarely that such quantities of new bone are formed in even children, as are commonly produced after necrosis of the shafts of bones in dogs or other animals; nor is there in the human subject any such filling up of the cavities from which superficial sequestra have been separated, as the experiments of Mr. Hunter showed, after such exfoliations from the metatarsal bones of asses.*

Other examples might be quoted; but these might suffice to show that, after injuries, new bone is formed more abundantly in animals than in man. And I hope enough has been said to prove that the generally-received account of provisional callus, and other parts of the healing of fractures, is an exaggeration of what occurs in man. It is to be asked what it is that is felt like a callus after fractures, I would say that, in such cases as I could examine after death, I have usually found that the overlapping ends of the bone, being both at once grasped, had been taken for the enlargement of callus. Sometimes, also, the thickening and induration of the parts around the fracture infiltrated with serous and bloody fluid, or with lymph, have been mistaken for it.

---

* Museum of the College, Nos. 641 to 653.

